# An estimation of airborne SARS-CoV-2 infection transmission risk in New York City nail salons

**DOI:** 10.1177/0748233720964650

**Published:** 2020-10-21

**Authors:** Amelia Harrichandra, A Michael Ierardi, Brian Pavilonis

**Affiliations:** 1 Department of Environmental, Occupational, and Geospatial Health Sciences, City University of New York Graduate School of Public Health and Health Policy, New York, NY, USA; 2 205740 Cardno ChemRisk, Brooklyn, NY, USA

**Keywords:** COVID-19, respiratory virus, droplet, infection control, pandemic, Wells–Riley equation

## Abstract

Although airborne transmission of severe acute respiratory syndrome coronavirus 2 (SARS-CoV-2) from person-to-person over long distances is currently thought to be unlikely, the current epidemiological evidence suggests that airborne SARS-CoV-2 infection transmission in confined, indoor spaces is plausible, particularly when outdoor airflow rates are low and when face masks are not utilized. We sought to model airborne infection transmission risk assuming five realistic exposure scenarios using previously estimated outdoor airflow rates for 12 New York City nail salons, a published quanta generation rate specific to SARS-CoV-2, as well as the Wells–Riley equation to assess risk under both steady-state and non-steady-state conditions. Additionally, the impact of face mask-wearing by occupants on airborne infection transmission risk was also evaluated. The risk of airborne infection transmission across all salons and all exposure scenarios when not wearing face masks ranged from <0.015% to 99.25%, with an average airborne infection transmission risk of 24.77%. Wearing face masks reduced airborne infection transmission risk to between <0.01% and 51.96%, depending on the salon, with an average airborne infection transmission risk of 7.30% across all salons. Increased outdoor airflow rates in nail salons were generally strongly correlated with decreased average airborne infection transmission risk. The results of this study indicate that increased outdoor airflow rates and the use of face masks by both employees and customers could substantially reduce SARS-CoV-2 transmission in New York City nail salons. Businesses should utilize multiple layers of infection control measures (e.g. social distancing, face masks, and outdoor airflow) to reduce airborne infection transmission risk for both employees and customers.

## Introduction

In the midst of the ongoing coronavirus disease 2019 (COVID-19) pandemic, an understanding of the potential route(s) of transmission of severe acute respiratory syndrome coronavirus 2 (SARS-CoV-2), the virus responsible for causing COVID-19, is of critical importance in the design and implementation of effective infection control measures. During the early stages of viral spread in the United States, infection mitigation strategies focused on viral transmission via fomites, or inanimate objects and surfaces that may carry infectious agents, such as door handles and elevator buttons. Large (>5–10 µm), virus-containing respiratory droplets emitted when infected individual coughs, sneezes, or talks, for instance, may contaminate a surface (WHO, 2020). Exponential decay of SARS-CoV-2 has been observed across different media, with estimated median half-lives of approximately <1 h on copper, <4 h on cardboard, 5.6 h on stainless steel, and 6.8 h on plastic (van Doremalen et al., 2020). Self-inoculation with SARS-CoV-2 could, therefore, occur if a susceptible (i.e. non-COVID-19-infected) individual touches a contaminated surface and subsequently touches the mucous membranes of their nose, mouth, or eyes (Otter et al., 2016; WHO, 2020). As such, initial recommendations consisted primarily of frequent handwashing as well as disinfection of high-touch surfaces with US Environmental Protection Agency (EPA)-registered disinfectants (EPA, 2020).

At the time of publication, however, the state-of-the-science as reported by the US Centers for Disease Control and Prevention (CDC) suggested that while adequate hygiene and disinfection are important, indirect transmission via fomites “is not thought to be the main way the virus spreads” (CDC, 2020c). Rather, a growing body of epidemiological evidence indicates that this novel human coronavirus is primarily spread from person-to-person via respiratory droplets or droplet nuclei, such that the risk of airborne SARS-CoV-2 infection transmission is likely highly dependent on both the duration of exposure and proximity to an infectious individual. Infectious respiratory droplets may land on the mucous membranes of a susceptible individual in close contact with an infected individual or may be inhaled by a susceptible individual in close proximity (CDC, 2020c). The CDC has defined “close contact” as being “within 6 feet of an infected person for at least 15 min starting from 2 days before illness onset (or, for asymptomatic patients, 2 days prior to positive specimen collection) until the time the patient is isolated” (CDC, 2020a). Indeed, many COVID-19 outbreaks have originated in indoor environments, including restaurants (Lu et al., 2020), churches (Yong et al., 2020), and cruise ships (Moriarty et al., 2020), where individuals are generally in close proximity with one another for extended periods of time and are talking, shouting, and/or singing—all activities that tend to produce respiratory droplets. Recommendations for universal (and proper) use of face masks and social distancing among the general public have proven effective in curtailing community spread of COVID-19 (Chu et al., 2020).

Yet these control measures may not be sufficiently protective to mitigate transmission risk via droplet nuclei shed by infectious individuals. Droplet nuclei are airborne residues (generally, ≤5 µm) of infectious aerosols from which the majority of respiratory fluid has evaporated (WHO, 2020). It has been demonstrated under experimental conditions that SARS-CoV-2 in aerosolized form may remain viable for up to approximately 3 h (van Doremalen et al., 2020); real-world evidence for airborne transmission of SARS-CoV-2 is still being gathered (Lednicky et al., 2020; Morawska and Cao, 2020). Given the currently available information regarding airborne transmission of SARS-CoV-2 and related viruses, however, it is reasonable to assume that SARS-CoV-2 transmission may occur if a susceptible individual inhales a sufficient quantity of viable droplet nuclei, though it is our understanding at the time this article was written that the infectious dose of SARS-CoV-2 above which there is a significantly increased risk of developing COVID-19 has not yet been established. Therefore, in addition to infection control measures like social distancing and face masks, attention must be given to ensuring adequate engineering controls in indoor environments (e.g. outdoor airflow), particularly in occupational settings where workers may be indoors for 8 h a day and interact with numerous individuals throughout the workday.

One example of an indoor, occupational environment where workers may experience prolonged contact with many individuals on any given day is the nail salon. Indeed, the American Industrial Hygiene Association (AIHA) has recently issued a COVID-19 guidance document specifically related to business reopening recommendations for nail salons (AIHA, 2020). We (AH and BP) have previously investigated indoor air quality issues at various nail salons in New York City. In a pilot study of 10 salons, total volatile organic compounds and carbon dioxide (CO_2_) concentrations were measured (Pavilonis et al., 2018), and we found that contaminant variation was generally minimal within each salon (i.e. well-mixed room). In a follow-up study, we estimated outdoor airflow rates per person using CO_2_ concentrations in 12 nail salons over three consecutive days and found little daily variation in airflow rates within salons; however, there were orders of magnitude differences in outdoor airflow rates between salons (Harrichandra et al., 2020).

Sufficient outdoor airflow is a critical precautionary measure when mitigating airborne infection transmission risk. As such, nail salons represent an important occupational setting in which airborne SARS-CoV-2 infection transmission risk for both employees and customers should be evaluated. New York City has more than 2000 nail salons that employ over 27,000 individuals (Basch et al., 2016). On July 6, 2020, New York City entered phase 3 of reopening, which allowed for the reopening of personal care services, including nail salons, with precautionary measures in-place (New York State Governor’s Office, 2020). As of this same date, there were approximately 216,000 cases of COVID-19 in New York City, with about 18,600 confirmed deaths and about 4600 probable deaths due to COVID-19 (New York City Department of Health and Mental Hygiene, 2020).

While three primary modes of transmission (contact via fomites, respiratory droplet transmission, and airborne [droplet nuclei] transmission) have been postulated during the COVID-19 pandemic, the focus of the current study is the risk of potential airborne transmission of SARS-CoV-2 in New York City nail salons. To estimate the risk of airborne infection transmission of SARS-CoV-2 in the confined, indoor spaces of New York City nail salons, the Wells–Riley equation can be utilized. This model was developed by Riley et al. (1978) to quantitatively assess the airborne risk of measles transmission during an outbreak in New York State in 1974. Riley et al. (1978) based their model on the “quantum of infection” concept first introduced by William Firth Wells in 1955 to signify the smallest dose of any infectious agent to cause infection in 63% of susceptible hosts (Wells, 1955). As explained by Rudnick and Milton (2003)

exposure to one quantum of infection gives an average probability of 63% (1 − e^−1^) of becoming infected (essentially an infectious dose 63%, ID_63_)…The belief that multiple independently deposited organisms are required to initiate infection is not borne out by biological evidence, nor is it biologically plausible. Thus *q* represents the generation rate of infectious doses, not organisms or infectious particles; it is the average infectious source strength of infected individuals. (Rudnick and Milton, 2003: 238)

The infectious dose of SARS-CoV-2 that may ultimately lead to COVID-19 development is unknown, but the infectious dose (LD_10_ and LD_50,_ respectively) for SARS-CoV-1 in animal studies was estimated to be 43–280 plaque-forming units (Watanabe et al., 2010). Using the average infectious dose coefficient (0.02) derived by Watanabe et al. (2010), the viral load of the sputum (10^9^ RNA virus copies/mL), and light exercise as the level of activity, the resulting quanta generation rate for SARS-CoV-2, as reported by Buonanno et al. (2020), was 142 quanta/h.

The objective of this study was to estimate the risk of airborne SARS-CoV-2 infection transmission in New York City nail salons under steady- and non-steady-state conditions using previously estimated outdoor airflow rates (Harrichandra et al., 2020).

## Methods

### Estimated outdoor airflow rate

We were unable to directly measure outdoor airflow rates. Therefore, we estimated outdoor airflow rates per person using Equation (6) from ASTM Standard D6245-18 and shown as Equation (1). The CO_2_ generation rate was selected for a female aged 21 to <30 years performing light work and 410 ppm was the average measured outdoor CO_2_ concentration (ASTM International, 2018). We multiplied the outdoor airflow rate per person by the number of workers and customers assumed to be in the salon at any given time based on logs provided by the salon owner. CO_2_ measurements were collected in each salon over a period of three consecutive days (Thursday, Friday, and Saturday) and averaged.

1 $$V_{o}=\left(\frac{N}{C_{S}-C_{O}}\right)\times 10^{6}$$

where *V_O_* = outdoor airflow rate per person (m^3^/s), *N* = CO_2_ generation rate per person (0.0000052 m^3^/s), *C_S_* = CO_2_ average concentration in the space (ppm), and *C_O_* = CO_2_ concentration in outdoor air (410 ppm).

As noted, outdoor airflow rates per person (m^3^/s-person) were previously estimated in 12 nails salons located in New York City (Harrichandra et al., 2020) and were used to calculate the risk of airborne SARS-CoV-2 infection transmission using the Wells–Riley equation. To calculate the total outdoor airflow rates (m^3^/min) in the nail salons (Table 1), the number of employees and customers were multiplied by the outdoor airflow rate per person.

**Table 1.** Nail salon characteristics.

	Salon											
	1	2	3	4	5	6	7	8	9	10	11	12
Volume (m^3^)	227.5	108.5	143.9	153.4	155.5	427.6	85.5	399	282.4	209.1	274.2	289
Outdoor airflow rate (m^3^/min)^a^	14.1	5.17	3.72	6.06	5.9	9.46	10.24	21.99	11.89	6.99	9.8	94.19
No. of occupants^b^	15	10	8	10	8	8	10	10	12	10	10	10

^a^ Adjusted for gravitational settling (*k*) and viral decay (λ).

^b^ Average number of customers and employees at any given time.

In addition to elimination through exhausted air, airborne droplets can be removed by viral inactivation (*l*) and gravitational settling (*k*). Viral inactivation refers to the chemical and physical changes in aerosolized viruses that result in loss of infectivity (Benbough, 1971). Buonanno et al. (2020) derived the value of *k* from a previously calculated settling velocity of particles that were approximately 1 µm (Chatoutsidou and Lazaridis, 2019). The diameter of SARS-CoV-2 particles ranges from 0.06 to 0.14 µm (Zhu et al., 2020). Viral decay was adopted from van Doremalen et al. (2020) based on the median estimate of SARS-CoV-2 half-life in aerosols of approximately 1.1 h. The values of *k* and *l* for virus removal were expressed as increased ventilation in the room with *k* being 0.24 air changes/hour (ACH) and *l* being 0.64 ACH. The number of ACH was multiplied by the volume of each nail salon and added to the total outdoor airflow rate.

### Impact of face mask use

The risk of airborne infection transmission can further be reduced by infected and susceptible individuals wearing face masks. In most public, commercial settings in New York City, social distancing and face mask-wearing orders have been enacted (e.g. New York State’s 10-Point PAUSE Plan and New York Governor’s Executive Order No. 202.17). For the purpose of this study, the term “face mask” generally encompasses N95 respirators, surgical masks, and homemade fabric masks or other face coverings. However, it should be noted that the efficacy of face masks depends on the type.

In fact, various forms of face masks have been found to reduce the transmission of respiratory viruses by 60% to 80%, and these viral transmission rates can be further reduced when face masks are worn in conjunction with adherence to social distancing protocols (Fennelly and Nardell, 1998; Liang et al., 2020; Nazaroff et al., 1998; Nicas, 1996). In this article, we use a conservative value of a 60% reduction in viral transmission from face mask use by an infected individual and expressed this transmission reduction as a 60% decrease in the quanta generation rate (*q*). To account for the reduction of transmission when a susceptible person is wearing a face mask, we also used the conservative value of 60% and expressed this as a 60% increase in the outdoor airflow rates (*Q*).

### Steady-state conditions

The probability of airborne infection transmission (*P*) in a room that has achieved a steady-state concentration is shown in the following equation (i.e. the Wells–Riley equation).

2 $$P\times 100=1-e\left(-\frac{Iq(\text{IR})t}{Q}\right)$$

where *P* = probability of airborne infection transmission, *I* = number of infected individuals (assumed as one [1] in this study), *q* = quanta generation rate (quanta/min), IR = inhalation rate (0.016 m^3^/min) (Buonanno et al., 2020), *t* = time (min), and *Q* = outdoor airflow rate (m^3^/min).

To calculate the risk of airborne infection transmission under steady-state conditions, the following scenarios were used:

1. *Scenario 1*: A susceptible employee is exposed to one infected employee for 480 min (8 h).
2. *Scenario 2*: At any given time, one susceptible customer is exposed to one infected employee for 60 min.

### Non-steady-state conditions

The traditional Wells–Riley model assumes steady-state ventilation conditions in which there is a constant generator of infectious particles (Riley et al., 1978). However, New York City nail salons do not meet this criterion if it is assumed that the generator of the infectious particles is a customer who briefly visits the salon and subsequently leaves after some time. Thus, the quanta concentration (*q_c_*) upon entrance to a nail salon by an infected individual was calculated using the following equation.

3 $$q_{c}=\frac{q}{Q}\left[1-e\left(\frac{-Qt}{V}\right)\right]$$

where *q_c_* = quanta concentration (quanta/m^3^), *q* = quanta generation rate (quanta/min), *Q* = outdoor airflow rate (m^3^/min), *t* = time (min), and *V* = volume of salon (m^3^).

Equation 4 was then used to estimate the decrease in quanta concentration (decay) when an infected individual exits the nail salon at *t*_2_.

4 $$q_{c2}=q_{c1}\times e^{\left[-\frac{Q}{V}(t_{2}-t_{1})\right]}$$

where *q_c_*_1_ = initial quanta concentration (quanta/m^3^) and *q_c_*_2_ = quanta concentration following decay (quanta/m^3^).

### Risk of airborne infection transmission under non-steady-state conditions

Quanta concentration (*q_c_*) was averaged over the scenario times and was used to calculate the risk of airborne infection transmission (*R*), as shown in the following equation.

5 $$R(\%)=100\times \left[1-e^{\left(-Ptq_{c}\right)}\right]$$

Three hypothetical exposures scenarios were used to calculate the risk of airborne SARS-CoV-2 infection transmission among employees and customers for non-steady-state conditions:

3. *Scenario 3*: One susceptible customer and one infected customer enter the nail salon together and both stay for 30 min.
4. *Scenario 4*: One infected customer enters and stays for 45 min, while one susceptible customer enters 30 min after the infected customer and stays for 60 min.
5. *Scenario 5*: One infected customer and one susceptible customer enter at the same time and both stay for 150 min (2.5 h).

### Statistical analysis

Pearson’s correlation coefficients (*r*) were calculated to evaluate potential associations between the outdoor airflow rate of each nail salon and the risk of airborne infection transmission, assuming with and without face mask-wearing, for all five exposure scenarios together, as well as for steady-state (i.e. scenarios 1–2) and non-steady-state (i.e. scenarios 3–5) conditions, separately. The normality of the estimated risk data was first assessed using the Shapiro–Wilk test for normality (null hypothesis [*H*_0_] = data are normally distributed). If the *p*-values for the Shapiro–Wilk test were greater than 0.05 for each scenario we assessed, then *H*_0_ was unable to be rejected and it was assumed that the modeled data were normally distributed. The statistical analysis was performed using SAS® software (9.4, SAS Institute Inc., Cary, North Carolina, USA).

## Results

The estimated outdoor airflow rates, adjusted for airborne virus removal from gravitational settling (*k*) and viral decay (l), are presented in Table 1. The average outdoor airflow rates across all salons were 16.63 m^3^/min and ranged from 3.72 to 94.19 m^3^/min. Salon 12 had the greatest outdoor airflow rate and relied on natural ventilation and did not have a dedicated HVAC system.

The risk of airborne SARS-CoV-2 infection transmission varied substantially across salons, particularly when accounting for use of face masks. The risk of airborne infection transmission across all salons and all exposure scenarios (i.e. under both steady- and non-steady-state conditions) when not wearing face masks ranged from <0.015% to 99.25% with an average airborne infection transmission risk of 24.77%. Additionally, wearing face masks resulted in an airborne infection transmission risk ranging from <0.01% to 51.96% with an average airborne infection transmission risk of 7.30%.

### Steady-state scenarios

When compared to airborne infection transmission risk calculated for similar exposure scenarios under non-steady-state conditions, the risk values derived using the Wells–Riley airborne infection transmission risk model under steady-state conditions were generally higher. Two exposure scenarios, assuming steady-state conditions, are compared in Table 2. These exposure scenarios are compared assuming neither an infected nor a susceptible individual were wearing face masks versus when both the infected and susceptible individuals were wearing face masks. The airborne infection transmission risk when wearing face masks was based on the assumption that both the infected and susceptible individuals were wearing face masks, which reflects current precautionary measures to be undertaken when utilizing personal care services in New York City, per New York State law, as noted above.

**Table 2.** Risk of airborne infection transmission (%) for two exposure scenarios, based on steady-state conditions, without (*N*) or with (*Y*) face masks.

**Salon**	** Scenario 1**		** Scenario 2**	
	***N* (%)**	***Y* (%)**	***N* (%)**	***Y* (%)**
1	72.44	17.58	14.88	2.39
2	97.02	40.96	35.54	6.38
3	99.25	51.96	45.71	8.76
4	95.00	36.21	31.24	5.46
5	95.40	36.99	31.94	5.61
6	85.34	25.03	21.34	3.54
7	83.04	23.38	19.89	3.27
8	56.24	11.66	9.820	1.54
9	78.30	20.48	17.38	2.82
10	92.56	32.29	27.74	4.76
11	84.35	24.30	20.69	3.42
12	17.54	2.850	2.380	0.36

**Figure 1.**
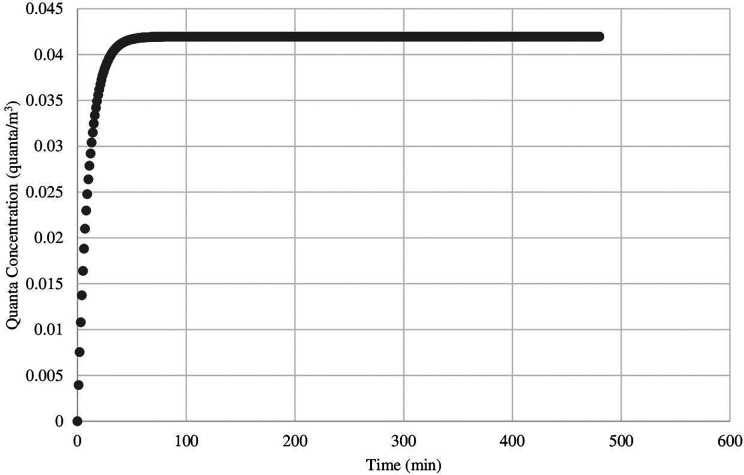
Illustration of quanta concentration increasing steadily and reaching steady state in scenario 1.

Across all nail salons, the risk of airborne infection transmission was greatest in scenario 1 in which a susceptible employee spends a full workday (8 h) with an infected employee. Wearing face masks resulted in a risk of airborne infection transmission that was generally much less than not wearing face masks for each salon. For example, the risk of airborne infection transmission in scenario 1 ranged from 17.54% to 99.25% when neither party were wearing face masks but decreased substantially to 2.85% to 51.96% when both parties wore face masks. Overall, there was an approximately 2- to 6-fold risk reduction in scenario 1 when face masks were worn. Furthermore, steady-state quanta concentrations were achieved between 25 min to 256 min across all 12 salons for scenario 1. In Figure 1, for example, steady state was reached in 118 min in salon 1.

### Non-steady-state scenarios

**Figure 2.**
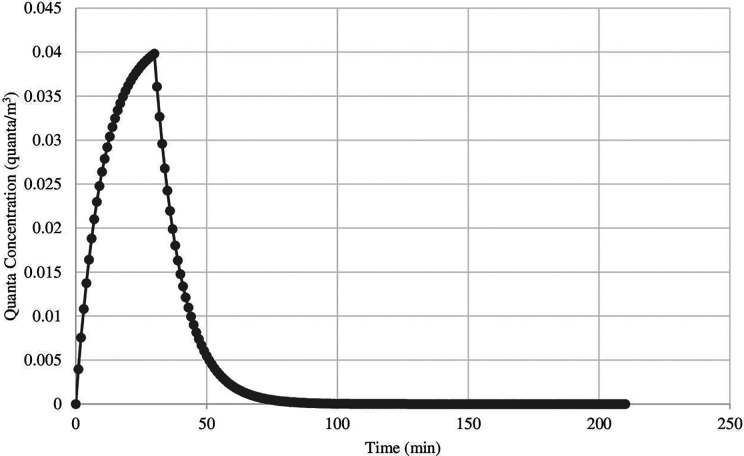
Illustration of quanta concentration decay as infected individual enters and then exits salon 1 (scenario 3).

Table 3 presents a comparison of airborne infection transmission risk under non-steady-state conditions for all salons when occupants (employees and customers) were not wearing face masks, compared to the airborne infection transmission risk when occupants were wearing face masks. As demonstrated in Figure 2, when the infected customer leaves the nail salon, the quanta concentration decreases and eventually reaches zero after 91 min, which is achieved at an outdoor airflow rate of 14.1 m^3^/min. Smaller nail salons with lower outdoor airflow rates typically had a higher risk of airborne infection transmission across all exposure scenarios evaluated. Salon 12 with an outdoor airflow rate of 94.19 m^3^/min had a risk of airborne infection transmission ranging from <0.015% to 17.54% (mean = 2.59%) across all five scenarios, while salon 3 with the lowest outdoor airflow rate of 3.72 m^3^/min had a risk of airborne infection transmission ranging from 1.47% to 99.25% (mean = 26.17%). Steady-state concentrations were reached fastest in salon 12 (25 min) and slowest in salon 6 (232 min), which had the highest volume (427.6 m^3^).

**Table 3.** Risk of airborne infection transmission (%) for three exposure scenarios, based on non-steady-state conditions, without (*N*) or with (*Y*) face masks.

**Salon**	**Scenario 3**		**Scenario 4**		**Scenario 5**	
	*N* (%)	*Y* (%)	*N* (%)	*Y* (%)	*N* (%)	*Y* (%)
1	4.27	1.35	0.68	0.04	7.690	1.98
2	9.71	3.25	3.26	0.26	19.58	5.31
3	8.84	3.19	9.83	1.47	25.47	7.28
4	7.43	2.53	3.83	0.37	16.91	4.54
5	7.43	2.55	4.18	0.43	17.31	4.67
6	3.17	1.14	4.42	0.73	10.71	2.91
7	7.69	2.24	0.10	0.00	10.43	2.72
8	2.59	1.04	0.70	0.23	5.000	2.03
9	4.02	0.94	1.78	0.01	9.020	1.28
10	5.79	2.01	4.17	0.48	14.75	3.95
11	4.36	1.49	2.75	0.30	10.79	2.84
12	1.06	0.28	0.00	0.00	1.190	0.30

In some exposure scenarios, the risk of airborne infection transmission was reduced substantially when wearing face masks. For example, in salon 1 for scenario 4, the risk of airborne infection transmission was reduced by 17-fold when a face mask was worn by both parties; however, in the same scenario for salon 3, which had the lowest outdoor airflow rate, the risk of airborne infection transmission was reduced more than 6-fold when a face mask was worn by both parties.

### Pearson’s correlation coefficients

The modeled airborne infection transmission risk data were all assumed to be normally distributed since the Shapiro–Wilk *p*-values for each scenario we assessed were greater than 0.05. In general, the outdoor airflow rates for each nail salon were negatively and strongly associated with airborne infection transmission risk (Table 4). In other words, as outdoor airflow rates increased within a nail salon, risk decreased. For example, for steady-state conditions (i.e. scenarios 1–2) assuming no use of face masks, there was a strong, negative correlation between outdoor airflow rate and average airborne infection transmission risk (*r* = −0.878; *p* < 0.001). Similarly, a correlation of *r* = −0.650 (*p* = 0.022) was calculated for non-steady-state conditions (i.e. scenarios 3–5) assuming no use of face masks.

**Table 4.** Pearson’s correlation coefficients (*r*) for nail salon outdoor airflow rates and airborne infection transmission risk.

Average risk (%)	*r*	*p* Value
Scenarios 1–5; no face masks	−0.833	<0.001
Scenarios 1–5; face masks	−0.681	0.015
Scenarios 1–2; no face masks	−0.878	<0.001
Scenarios 1–2; face masks	−0.690	0.013
Scenarios 3–5; no face masks	−0.650	0.022
Scenarios 3–5; face masks	−0.620	0.031

## Discussion

The objective of this study was to estimate the airborne infection transmission risk of SARS-CoV-2 among employees and customers in nail salons in New York City as businesses reopen in the wake of the pandemic. Previously published outdoor airflow rate data (Harrichandra et al., 2020) and a quanta generation rate for SARS-CoV-2 (Buonanno et al., 2020) were used in the Wells–Riley model to assess the risk of airborne infection transmission under various hypothetical exposure scenarios characterized by the interaction of employees and customers in nail salons in New York City. The modeled data indicate that adequate outdoor airflow rates and the use of face masks by both employees and customers could substantially reduce the risk of airborne SARS-CoV-2 transmission in New York City nail salons.

In New York City, many nail salons have adopted the CDC’s guidelines for protecting employees and customers, such as practicing social distancing through a reduction in the capacity of services to fewer customers at any given time, removing waiting areas and accepting customers by appointment only, installing Plexiglas between service stations, and requiring all employees and customers to wear face masks at all times (CDC, 2020b). The results of this study indicate that increased outdoor airflow can reduce the risk of airborne infection transmission. For example, salon 3 had the lowest outdoor airflow rate (3.72 m^3^/min) among all of the salons and, subsequently, the highest risk of airborne infection transmission across both steady-state (scenario 1 = 99.25%) and non-steady-state (scenario 5 = 25.47%) scenarios, when no face mask-wearing was assumed. In comparison to salon 12, which had the highest outdoor airflow rate (94.19 m^3^/min), the risk of airborne infection transmission was the lowest among both steady-state (<17.54%) and non-steady-state (<1.19%) scenarios, when no face mask-wearing was assumed. It should be noted that salon 12 utilized natural ventilation and did not have a dedicated exhaust. While this method of control is feasible in the summer months, this would not be effective in colder months. In a similar study focusing on the role of ventilation in the spread of COVID-19, it was concluded that reducing occupancy by 50% reduced the risk of airborne infection transmission by 6.7% based on a 90-min exposure duration in a restaurant, with similar dimensions to the nail salons; however, it was also demonstrated in this study that increasing the ventilation rate by approximately 27% could achieve the same rates of airborne infection transmission risk reduction (Sun and Zhai, 2020).

In the steady- and non-steady-state scenarios, worst-case and best-case scenarios were primarily determined by exposure time to an infected person. In scenario 3 in which two customers, one infected and one susceptible, enter the salon at the same time and both stay for 150 min, the airborne infection transmission risk increases substantially until the infector leaves but does not immediately drop to zero. In scenario 4 in which an infected customer enters the salon and stays for 45 min, while one susceptible customer enters 30 min after the infected customer and stays for 60 min, the risk of airborne infection transmission was still high and ranged from >0.01% to 9.83% across salons. This finding may explain why the SARS-CoV-2 virus spread so quickly initially in densely-populated cities around the world and should be a consideration as businesses reopen to the public. Merely permitting fewer customers may not sufficiently reduce the risk of airborne infection transmission without increasing the amount of outdoor airflow. If outdoor airflow remains the same, the rate at which customers enter the salon can be reduced so that fewer customers are in the salon when the concentration of infectious materials is at its highest, before concentration decay begins. This can be achieved through appointments that stagger the arrival of customers over a given time.

The role of face mask-wearing was heavily contested at the onset of the pandemic but is now accepted as an efficacious measure to reduce the spread of COVID-19 (Feng et al., 2020; Lyu and Wehby, 2020; Ngonghala et al., 2020). The results of this study demonstrated that a face mask worn by both infected and susceptible parties could substantially reduce the risk of airborne infection transmission, even when outdoor airflow rate was poor and the duration of exposure was long. In the worst-case scenario of two employees, one infected and one susceptible, spending a full workday together and assuming that no other infected person enters the salon (i.e. scenario 1), the risk of airborne infection transmission of the susceptible employee was reduced from an average of 79.71% when neither parties wore a face mask to 26.97% when both parties wore a face mask, an almost 3-fold reduction in risk. Further, in salon 3, which had the lowest outdoor airflow rate, wearing face masks reduced the risk of airborne infection transmission by 47.29% for scenario 1. In a recent study of COVID-19 transmission in a hair salon, where two symptomatic, COVID-19-positive hair stylists served 139 clients, all wearing masks, over 15- to 45-min periods (mean = 19.5 min), there were no reported positive cases within a 14-day period (Hendrix et al., 2020).

One study estimated that had New York State met 100% face mask compliance on the first day of the shelter-in-place order, the cumulative mortality rate from COVID-19 could have been four times less; even a 50% compliance rate could have halved the number of deaths recorded (Ngonghala et al., 2020). Since SARS-CoV-2 can be transmitted via droplets during close contact, any face covering, including homemade cloth masks and surgical masks, that traps exhaled droplets can reduce the amount of infectious airborne particles emitted as well as the amount that can be inhaled by a susceptible individual.

It is acknowledged that there are still gaps in the literature regarding the transmission of this novel human coronavirus. The value of the quanta generation rate (*q*) has varied among a few studies (Buonanno et al., 2020; Dai and Zhao, 2020; Zemouri et al., 2020) and needs to be studied further. The value of *q* used in this study was derived from a novel approach based on the viral load emitted in saliva (Buonanno et al., 2020). Yet there may be more accurate values based on other approaches. In this study, we used a conservative value for the quanta reduction potential of face masks based on several studies. The risk of airborne infection transmission may vary significantly from the modeled results presented in this study when different types of face masks are utilized in different settings. In addition, we assumed one infected individual was present in each of the exposure scenarios. Future research should evaluate airborne infection transmission risk assuming multiple infected individuals are present in a confined space for a given period of time.

## Conclusions

This study found that adequate outdoor airflow and adherence to wearing face masks can reduce the risk of airborne SARS-CoV-2 infection transmission in New York City nail salons. Increased outdoor airflow has the potential to reduce the risk of airborne infection transmission to approximately <1% when face masks are worn by all occupants of a confined space. Social distancing and reduction of contact time are also essential to reducing the risk of airborne infection transmission. As New York State continues to gradually reopen, it is imperative for individuals to continue observing social distancing and face mask-wearing requirements and for establishments to ensure that buildings are properly ventilated and are not overcrowded to mitigate potential airborne SARS-CoV-2 infection transmission risk.
